# Center for Behavioral Neuroscience: a prototype multi-institutional collaborative research center

**DOI:** 10.1186/1747-5333-1-9

**Published:** 2006-07-17

**Authors:** Kelly R Powell, H Elliott Albers

**Affiliations:** 1 Center for Behavioral Neuroscience, Georgia State University, P.O. Box 3966, Atlanta, Georgia 30302-3966, USA

## Abstract

The Center for Behavioral Neuroscience was launched in the fall of 1999 with support from the National Science Foundation, the Georgia Research Alliance, and our eight participating institutions (Georgia State University, Emory University, Georgia Institute of Technology, Morehouse School of Medicine, Clark-Atlanta University, Spelman College, Morehouse College, Morris Brown College). The CBN provides the resources to foster innovative research in behavioral neuroscience, with a specific focus on the neurobiology of social behavior. Center faculty working in collaboratories use diverse model systems from invertebrates to humans to investigate fear, aggression, affiliation, and reproductive behaviors. The addition of new research foci in reward and reinforcement, memory and cognition, and sex differences has expanded the potential for collaborations among Center investigators. Technology core laboratories develop the molecular, cellular, systems, behavioral, and imaging tools essential for investigating how the brain influences complex social behavior and, in turn, how social experience influences brain function.

In addition to scientific discovery, a major goal of the CBN is to train the next generation of behavioral neuroscientists and to increase the number of women and under-represented minorities in neuroscience. Educational programs are offered for K-12 students to spark an interest in science. Undergraduate and graduate initiatives encourage students to participate in interdisciplinary and inter-institutional programs, while postdoctoral programs provide a bridge between laboratories and allow the interdisciplinary research and educational ventures to flourish. Finally, the CBN is committed to knowledge transfer, partnering with community organizations to bring neuroscience to the public. This multifaceted approach through research, education, and knowledge transfer will have a major impact on how we study interactions between the brain and behavior, as well as how the public views brain function and neuroscience.

## History of the Center for Behavioral Neuroscience (CBN)

The Center for Behavioral Neuroscience was originally funded in 1999 as part of the National Science Foundation's (NSF) Science and Technology Center (STC) initiative. The STC program was designed in the late 1970s to enable innovative research and education projects of national importance that require a Center mode of support to achieve the research, education, and knowledge-transfer goals shared by the partners. STCs conduct world-class research in partnerships among academic institutions, national laboratories, industrial organizations, and/or other public/private entities to create new and meaningful knowledge of significant benefit to society. In addition, STCs build intellectual and physical infrastructures within and between disciplines, and foster education by integrating education with research, and by creating bonds between learning and inquiry so that discovery and creativity more fully support the learning process. In summary, NSF's Science and Technology Centers offer the research and engineering community an effective mechanism to undertake long-term scientific and technological research and education activities, to explore better and more effective ways to educate students, and to develop mechanisms to ensure the timely transition of research and education advances made into service in society.

In the late 1990s, a small group of faculty from Emory University and Georgia State University began to develop an outline for a new center that would promote research and education in the area of neuroscience involving eight Atlanta-based academic institutions. These institutions were selected based on their potential to lend themselves to the research and educational missions of the center and on their geographical location. One of the major initial challenges was to define a research focus that would build on the research strengths of the neuroscientists in Atlanta. A series of retreats and meetings were held where it was determined to build the research focus on existing strengths in behavioral neuroscience with an emphasis on aggression, affiliation, fear and reproduction. Following a year focused on the development of proposals and a series of evaluations culminating in a comprehensive, two-day site visit by the NSF, the center was one of five STC centers chosen for funding from several hundred other initial applications. The CBN was chosen to receive one of the ten-year awards of approximately $40 million beginning in November of 1999. In addition, the Georgia Research Alliance (GRA), a private, nonprofit corporation whose mission is to capitalize on innovative university research to build a vibrant, technology-rich economy pledged to support the Center with an additional $16 million. The funding from the GRA has been critical to the recruitment of new faculty into the center's institutions and acquiring necessary equipment for new technologies used for the center research.

## Organizational structure of the CBN

**Figure 1:**
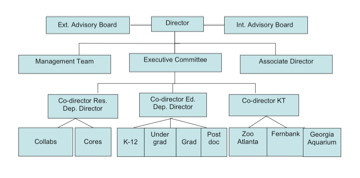
Organizational Chart of the Center for Behavioral Neuroscience.

The organizational structure of the CBN is best illustrated in the diagram below (figure 1). The Director of the center receives advice from two key advisory Boards that meet annually (these are not voting or policy-making entities). The Internal Advisory Board is comprised of the Provosts from each of the eight partner institutions (or a representative of the Provost) and provides guidance on issues related to the basic operations of the center as they pertain to the operations of the individual partner institutions. The External Advisory Board in comprised of 12–13 scientists, educators and professionals from outside the center and provides guidance on the scientific and educational aspects of the center. The director is assisted by three co-directors who oversee the directions and programs in research, education, knowledge transfer, and by an associate director who oversees the daily operations of the center.

The co-director for research, who is assisted by a deputy director, works with a number of faculty in the center who lead "collaboratories" that are based on thematic research topics and technology "cores" focused on the development of new technologies for behavioral neuroscience research. The "collaboratories" consist of a group of investigators from different laboratories and institutions who come together to discuss common research interests. These groups meet in real time (using video-conferencing technology to bring together investigators from the different institutions) to foster discussion and collaboration among investigators and to provide expertise to investigators among whom there may be no collaborations, but where there is common interest in progressing the research field. Together, with input from the membership as a whole, the co-director, deputy director and collaboratory heads determine what research should be supported directly by the center and work to encourage research in areas that they believe will best benefit the field in general. These become the research topics that are the focus of the "collaboratories." The co-director for education is assisted by the deputy director for education who oversees the daily operations of the center's various educational programs and assessment of these programs. The co-director for knowledge transfer oversees the development of community partnerships and the technology transfer issues of the center. Additional staff include the director of information technology, who oversees the development and maintenance of various technologies used by the center, the communications coordinator, who oversees public relations, advertising and some development for the center, and the financial business manager, who manages the center's finances. A host of administrative assistants, faculty-led committees and volunteers also work to make the centers research, educational and community partnership activities possible.

Membership in the CBN is based primarily upon interest on the part of the candidate to contribute to and benefit from the unique resources that the center provides. Membership in the CBN involves an application and approval process led by specific committees. Members should be committed to furthering the field of behavioral neuroscience in some manner consistent with the CBN's mission, and the only other requirement of membership is for individuals to provide information on their own research and educational activities for the center's annual report. Current membership in the center includes nearly 100 faculty, 20 postdocs and 50 graduate students representing all our member institutions, with the larger research-intensive institutions making up a significantly larger part of the membership.

## Main goals and objectives of the CBN

The CBN began its tenure with a broad vision to become a national resource for the field of behavioral neuroscience, contributing new knowledge, training a diverse student population, and bringing an appreciation of science to the public at large. To this end the center's official mission statement is: **The CBN mission is to bring together the unique resources from a consortium of Atlanta colleges and universities to build a nationally recognized program that will (a) define the interaction of brain processes and complex behaviors, (b) create a cadre of interdisciplinary investigators focused on behavioral neuroscience, and (c) transfer relevant discoveries from the laboratory to the public**. With such a broad vision, one of our biggest challenges has been to develop a strategic plan that will promote our mission for research, education and knowledge transfer without diluting the efforts in any of these three areas.

### Research goals and objectives

The primary goals of our research initiatives include 1) developing a model of collaborative research among investigators using diverse techniques, levels of analysis, and model systems, 2) adding new knowledge about the neural systems underlying complex behavior, and social behavior in particular, and 3) enhancing the neuroscience research infrastructure at all participating institutions. These goals have been broken down into more specific goals and objectives, each of which we consider key to promoting the overall mission of the center.

The center's original research structure involved focusing on four primary research themes: affiliation, aggression, fear, reproduction. These research themes are the focus of the research conducted within the "collaboratories," which, as previously mentioned, act as conduits for establishing inter-institutional and inter-laboratory research collaborations to promote innovative research within these specific theme areas. As the science in these areas progressed it became evident that we needed to broaden and enhance the collaborative, multi-disciplinary interactions in the center to bring in new ideas and identify new avenues for research, something that we expect will need to be repeated over time. Through the collaboratory structure, we have initiated cross-collaboratory activities focused on new themes cutting across all aspects of social neuroscience to supplement and enhance on-going collaboratory activities. These cross-collaboratory themes include sex differences, reward and reinforcement, and cognition and memory. Not only has this pushed the science into new, exciting directions, but it has also attracted more scientists into the center, providing new opportunities for research collaborations. For example, research supported by the CBN resulted in the finding that social bonding in voles has a specific genetic basis, a result reported by over 140 official media sources. Research supported by the CBN has also resulted in the development of a new, effective treatment for certain phobias. These are only two examples of some of the cutting-edge research studies that have resulted from the center's unique structure of resources. Select studies from the past two years are listed in the References section to provide a broader picture of the type of behavioral neuroscience research supported by the center [1-73]. Most of these selected studies involved graduate scholars and postdoctoral fellows as key investigators. Research supported by the center can also be accessed on the center's website [74].

A significant challenge for our center has been adding new knowledge about social behavior and neuroscience to the field while developing and/or maintaining vigorous research programs at all the partner institutions, including those institutions whose missions are not research intensive. By encouraging participation of investigators from all the participating institutions in the collaboratory activities, we have created a community that provides resources and intellectual support for all investigators, most notably for those investigators who may have been conducting research in relative isolation prior to joining the center. In addition, the center provides technical support through our technology cores to all center investigators who wish to expand their research to include the new technologies developed by the cores. Finally, our venture grant program provides funding for innovative, collaborative research projects that can especially benefit investigators who do not have other sources of funding.

Another research mandate for our center is to enhance the research infrastructure at participating institutions. Early in the life of the center, it became evident that the unique missions of the partner institutions had to be directly addressed before addressing the center's research objectives with regard to these institutions. To this end, a task force, consisting of representatives from each of the partner institutions, was put together to more effectively address the individual institutional missions while promoting the center's research objectives. The task force was faced with unique challenges and took a number of approaches to meet these challenges. Obviously, some of our participating institutions have neuroscience research as a priority in their own missions and we simply try to capitalize on their efforts by offering incentives from our center to attract new hires in behavioral neuroscience to these institutions. Other institutions did not originally consider this as a priority for their missions, but interestingly, we found that by using center resources to help these institutions with their educational priorities, some of them have begun to incorporate more emphasis on neuroscience education and research into their missions. By working with individual departments and leveraging the center's funding from the Georgia Research Alliance, the CBN has been directly involved in the hiring of over 30 neuroscience faculty across the partner institutions, including fourteen female faculty. The key has been to join each institution (and even individual departments) in promoting their missions (where there was mutual benefit to the center's mission) and allowing the leaders at each institution observe for themselves the benefits of partnering with our center in order to obtain "buy-in" from our partner institutions.

### Educational goals and objectives

Key educational goals of the center include 1) training graduate and postdoctoral students in behavioral neuroscience, stressing interdisciplinary, collaborative research, 2) increasing interest in neuroscience research careers at the undergraduate level by providing opportunities for intensive research experiences for those students, 3) improving science education at the K-12 level and stimulating interest in science in those students, and 4) increasing the number of underrepresented minorities and women entering scientific careers.

The collaboratory structure of the center provides a unique training environment for center postdocs, graduate and undergraduate students. This environment exposes postdocs and students to investigators from multiple disciplines, allows for training in multiple environments including training with new, cutting-edge techniques for research, and promotes techniques of collaboration in research by encouraging all postdocs and graduate students to be involved in collaborative research projects across labs and even across institutions. Postdocs and graduate students leave the center with a broad training and better prepared for the latest movements in collaborative, multidisciplinary neuroscience research. As postdocs and graduate students leave the center, they take this innovative approach to research into new places and positions in the field.

Undergraduate students who work in center-affiliated research labs are mentored in ways that will encourage them to consider strongly pursuing a graduate education in neuroscience. To this end, it is key that these students feel a part of the community of scientists and that they gain confidence in themselves as part of a community of scientists. This includes providing these students with a meaningful laboratory research experience where they feel they are an important part of the research process and helping them to develop skills and confidence in their own intellectual abilities so that they will be able to see themselves in scientific careers. Undergraduate students are encouraged to participate in collaboratories and in individual laboratory meetings. In addition, the center provides some funds to send some of the best students to national meetings to present their research. Providing younger students mentorship from graduate students and from upper level undergraduates in the community can also encourage these students into the community.

K-12 educational outreach should not be overlooked when building the future of a large scientific center. K-12 educational outreach can be very attractive to potential financial supporters, from foundations to individuals in the non-scientific sector. The main purpose of K-12 educational outreach is to address the idea that students often need to develop a love of science early in order to be ready and willing to take advantage of the opportunities afforded them once they reach college. A healthy love of science depends in large part upon K-12 science teachers and how they portray and promote the topic to children in their classes. Our center provides week-long teacher training workshops that provide teachers with new information and tools for teaching science to their students and with practical skills for making their teaching more interesting to students. In addition, our center offers summer camps and research programs for middle and high school students to try to attract them to science as they begin to develop notions of what careers they might pursue.

Attracting underrepresented minorities into neuroscience remains a challenge for nearly every institution nationwide. Through our undergraduate research programs, many minority students at the center's partner institutions have participated in the ongoing research in our center; however, we cannot depend on students from our own partner institutions to meet our goal for increasing minorities in neuroscience.

### Knowledge transfer goals and objectives

The primary thrust of the CBN's knowledge transfer element is to transfer relevant discoveries from the laboratory to the public. Because the CBN's research is predominantly basic science rather than technology development, the center's knowledge transfer typically takes the form of public education and a major goal is for the CBN to become a leader in public education about the social behavior of animals and humans and related topics.

Original efforts at public education included a few poorly attended public lectures. It was soon evident that the CBN did not have the marketing infrastructure to attract the lay public to these sorts of activities. Since then, the CBN has developed a different approach to public education that revolves around partnerships with local organizations that have similar goals, including Zoo Atlanta, the Georgia Aquarium and the Fernbank Museum of Natural History. Through these partnerships, the center sponsors events that bring neuroscience to the public in meaningful ways. These events include an annual neuroscience exposition at the zoo that features several fun, interactive booths that instruct visitors about animal and human behaviors. Although it was not initially evident how our center could partner with a natural history museum, through a series of discussions with the staff of the museum we were able to design unique ways to educate the public through this partnership. For example, we have featured film screenings of popular movies at the museum, followed by discussions led by center scientists about the human social behavior issues featured in the movies. In addition, the museum featured a temporary exhibit on the topic of genomics for which our center trained docents and provided wet lab demonstrations to help educate the public on this topic. These types of events put the public in direct contact with scientists from whom they can learn about science and have their questions answered.

The CBN has also established partnerships with other neuroscience programs nationally through student and postdoc exchanges to promote the exchange of ideas and resources for furthering the reach of the center's resources nationally.

The internet and other media outlets provide excellent tools for providing information about the center and its research and educational programs to center members and the general public. Creation of a user-friendly and informative website has been helpful in marketing the center and its programs nationally. The CBN staffs a fulltime communications coordinator who oversees the marketing of the center both locally and nationally. Through press releases about the center's science and programs, the CBN's name is becoming more recognized among other behavioral neuroscientists and among some non-science audiences.

## Examples of programs designed to meet center objectives

A Venture Grant Program was established from the start of the center, designed to support high-risk, innovative research projects that would be difficult to obtain funding for through traditional grant mechanisms. The main purpose of the program is to fund new investigators in behavioral neuroscience and to provide seed money (up to $30,000 per grant) for multidisciplinary, collaborative research projects that will push the field of behavioral neuroscience forward into new directions. In order to help meet the center's research and education goals, funded research projects must be collaborations among two or more center investigators, must aim to provide important information to the respective field of research, and must include some educational component such as the training of undergraduate students, graduate students and/or postdocs. Initially, the venture grant program was run like a smaller version of the federal grant programs, funding a broad spectrum of research proposals in the area of behavioral neuroscience. This, however, did little to promote more innovative research that would push the field ahead. This approach was changed early on and the research focus of the center became more narrow and placed under the four initial collaboratory themes: affiliation, aggression, fear, reproduction. Through the collaboratory structure, research funded by the center could be prioritized to create a portfolio of research projects that were more effective at pushing ahead specific areas of behavior neuroscience. One critical requirement of the venture grant award is that the research projects funded be collaborative. This creates a community of collaborators each of whom can bring their expertise to the table to enhance the creativity and innovation of the research conducted. Also, the program establishes a mentoring process through which more seasoned investigators can advise and guide newer investigators to produce more successful research projects and successful investigators. Rarely is any venture grant rejected outright. Those proposals that are not funded in one round of awards are usually recommended for resubmission following specific changes and careful review of the proposal by the members of the collaboratory and the collaboratory head, who acts as a mentor in this regard. When warranted, results from venture grant projects are used to obtain grants through other mechanisms. Thus far, the CBN's Venture Grant Program boasts funding of 84 collaborative research and educational projects in six years. Venture grant funding distribution has been roughly proportional to the number of faculty investigators at each institution. This is due in large part to the encouragement and guidance that the research faculty from each institution receives through the collaboratory structure. Within this structure, venture grant proposals are presented and enhanced by guidance from the larger group and the collaboratory's head before the proposals are submitted for formal review by the venture grant approval committee. To date, the CBN has invested approximately 1.8 million dollars in this venture grant program, which has seeded grants awarded from other sources in the amount of approximately 35 million dollars.

The center's Graduate Scholars Program was designed to establish a community of graduate students from different graduate programs and different institutions through which professional relationships could be forged and research collaborations could be initiated. Using some of the NSF funding, the center has attracted over 50 students into participating graduate programs by offering full or partial stipends and some funding for travel and supplies to these students. Participating students are required to take classes together and to attend center seminars, among other center-wide events, where the sense of community is strengthened. Over the first six years, at least 30 of the venture grants awarded involved graduate students from different laboratories working together on a single research project. In addition, center graduate students take classes together taught by faculty from the different institutions and have the opportunity to develop professional relationships with faculty in other neuroscience programs and other institutions. Many of the students have invited faculty from other programs and institutions to be part of their dissertation committees, thereby gleaning expertise from faculty outside their own programs. Clearly, this program has provided a much broader community of professional neuroscientists from which center graduate students can draw for their education and professional growth. Moreover, these students establish working relationships with peers outside of their own graduate programs, which will broaden their professional contacts and, perhaps, increase their motivation and skills to collaborate with other scientists in the future. This graduate program has been especially successful in attracting female and minority graduate students (higher percentage of female and minority graduate students than the average neuroscience program as indicated by annual ANDP reports). It is possible that some of the success is due to our close relationships to local historically black colleges and universities and some to the positive environment the center tries to provide to women and minorities. In addition, graduates from our center have all been placed in either postdoctoral positions at major research institutions or tenure track positions in teaching and/or research.

Public education about the center's research and about neuroscience in general has been a big challenge requiring creative methods. The center's relationships with the local zoo, aquarium and museum of natural history provide more access to public audiences in venues where people are open to learning. Annually, the CBN co-sponsors with Zoo Atlanta a Neuroscience Exposition and Brain Fair. Not only does this event bring an air of extra excitement to the zoo for the day, but it also increases the exposure of the zoo's visitors to neuroscience research and the scientists who conduct the research in a fun environment. The center puts together 20–30 interactive booths that are placed strategically around the zoo and manned by center volunteers including graduate students, postdocs and faculty. The booths are designed to teach very general concepts about animal behavior and the brain and to allow the general public access to scientific information and scientists to which they don't typically get access. Each year has seen increased attendance to this event and our surveys from the events indicate a very positive response by the public to the event and the information presented. In addition, the CBN gains important local recognition, which can help in obtaining future support, both financial and other.

These are only a few examples of the successful programs launched by the CBN that have impacted not only the information we have gained about the brain and social behavior, but also the manner in which this information is gathered through scientific research. Moreover, these programs have aided us in making strides toward a public more informed about the brain, behavior and the research process.

## Intra- and inter-institutional challenges

Breaking with traditional models of education and research is expected to bring many challenges. The CBN has faced many challenges in establishing its collaborative, inter-institutional model of conducting research and educating students and postdocs, some of which continue to hinder progress toward our ultimate goals.

Perhaps the largest challenge for our center has been to maintain the lines of communication with institutional leaders, including presidents, provosts, deans and department heads, needed to foster the center's mission. Working with several different administrations, each with its own organizational methods, can be a huge challenge for accomplishing even simple things. For example, new courses typically must be formally presented and approved by the appropriate administrators at each institution before being added to the curriculum. Rather than go through this tedious process at each institution, the center offers courses as "general topics" courses. Most departments/programs have at least one course listing under which a "general topics" course may be offered for credit. If sponsored by at least one faculty member in that department/program, the center course can be offered for credit to students in that program for a given semester. Although challenges such as this may not seem terribly critical or even difficult to solve, when you have several of these types of challenges at once, each simple challenge can seem monumental to solve.

One example of an ongoing challenge is developing a system by which faculty in different departments and institutions can get tenure-related credit for the work they do for the center. It is expected that departments and institutions put their own needs and goals first and not allow outside organizations to distract from meeting those needs and goals. However, if the center's goals are aligned with the goals of a department and institution and can help those entities meet their needs and attain their goals, allowing faculty to participate in center work could be seen more favorably and even counted towards tenure and institutional service. Therefore, CBN leaders strive to work alongside department and institutional leaders to determine how the center might best help them achieve their goals without compromising the center's mission. It is hoped that this will ultimately lead to greater support for the center and its activities from such leaders and to more symbiotic relationships between and among the departments and institutions from which the center draws its membership. Nonetheless, developing this support remains challenging in the face of so many other competing interests at the institutional and even departmental level. Regular interaction with administrative leaders is required to maintain an alignment with the needs and goals of several different entities while also promoting the center's needs and goals. Initially, the CBN proposed to use its Internal Advisory Board, composed of institutional provosts, to create and maintain this regular interaction; however, many of these leaders changed so frequently that it was difficult to maintain any institutional memory of and continuous support for the center and its mission. Thus, this issue remains one of our chief challenges.

Another critical challenge for our center has been the development of a strategic plan that will not only meet the requirements of our primary funding source (the NSF), but will also create a sustainable research and education center that can attract financial and other support to sustain itself once the primary funding is gone. Our center was initially awarded five years of funding with the potential for five additional years if the center was successful in meeting the goals set before us by the NSF. The strategic plan for the first five years focused solely on meeting NSF goals for the center so that an additional five years of funding would be awarded. Currently we are in the second five years of our award and we are faced with the challenge of how to reinvent the center in order to sustain it once the NSF funding is gone. This requires the development of a completely different strategic plan and making the difficult decisions about which, if any, of the programs we have worked so hard to develop will have to be dropped in order to focus on programs that will attract necessary funding. In addition, there are a variety of opinions about what the center should become and what programs should be maintained or dropped, even among the center's leadership. Moreover, the process of creating a long-term, sustainable strategic plan requires a lot of energy and thought from people with concerted interest in the center. After several years of putting forth effort that is additional to primary duties at their institutions, some faculty members of the center have naturally become focused on other priorities. This has presented a serious challenge to the survival of the center. Even though there are over three years remaining from our primary source of funding, it is evident that engaging in the strategic planning for the center's future cannot be done too early. In fact, it has become clear that this process should begin as soon as a center is initiated. Given that institutional goals and missions often change, especially for those institutions engaged in scientific research, it should be expected that any institution or center focused on promoting any area of scientific research and education remain actively engaged in reevaluating its goals and strategic plan to best meet the needs of the field it serves.

To be certain, there are many other challenges that face an inter-institutional research and education center that draws from several different institutions for its leadership and general membership. Creating this type of center requires regular interaction with its institutional members in order to develop and maintain the support it requires from these institutions. Moreover, long-term sustainability of any center requires constant re-evaluation of its goals and strategic plan for meeting those goals.

## Assessment and evaluation

Assessment is critical for the success and sustainability of our programs; however, assessment of the many and varied programs begun by our center has been an administrative challenge, particularly with the educational programs. It has become clear to us that a key need for our center is identifying the best methods to assess our variety of programs that are intended to meet the goals of the center. The original strategic plan for the center did not include a comprehensive plan for assessing its programs. Since then, we have worked toward finding or developing the best types of assessment to provide the information we want about the impact the center has in many different areas. Each program must have defined goals and objectives and specific methods for meeting those objectives and goals. In addition, the measures for determining the success of meeting the objectives and goals must be measurable, reliable and valid. Once assessment plans are developed, collecting data can be a daunting undertaking. We have developed a customized online database for storing and retrieving data about our programs as needed. Data is entered as it is collected and then retrieved in a format that can be used for assessment of different programs or for reporting purposes. This database has become a key player in the assessment process for all of the center's programs.

Our center must constantly reevaluate the methods we use to meet our objectives and goals in order to ensure efficiency, both financial and temporal. By methods we mean use of staff and center members, use of particular administrative methods, use of programs and use of other factors for the purpose of promoting our mission. These reevaluations have stressed the importance of keeping our methods flexible. That is, when inventing a new way of doing something, one must be flexible enough to change methods as needed to ensure success. For our center this includes hiring staff that understand that their job responsibilities will likely change over time and who will be able to change their skills with the changing responsibilities. Depending upon the strategic objectives for the center at a given time, different administrative needs will arise and new methods of meeting those needs must be adopted. For example, in our second five years, we have needed to focus much more on public relations and name recognition for our center in order to develop a reputation that will attract future funding. This has necessitated a change in the focus of at least one of the center's full time staff persons from education to public relations and development. In some cases, this might require a change in staff to meet these specific administrative needs. Also, the center members have changing priorities in their professional lives that necessitate that the center be flexible in what it asks of its members. Finally, the center has to be willing to alter its programs in order to meet changing objectives and goals over time. This might simply require redesigning a program to better meet primary objectives and goals or this might require completely replacing or dropping certain programs to better meet the objectives and goals of the center as a whole. The process of evaluation of the center's methods for meeting objectives and goals is no small task and requires full time administrative oversight, usually by someone who is familiar with all aspects of the center and is able to focus on how all the different parts of the center are collectively meeting the objectives and goals of the center.

## Financial support for a growing, changing center

Critical factors for obtaining financial support for a large research center are very similar to those required for smaller endeavors. These critical factors include 1) development and maintenance of a valid, workable strategic plan that meets the needs of the field served, 2) assessment and redesign of programs to ensure their successful impact on the audiences served, and 3) regular reevaluation of the methods used to meet center objectives and goals top ensure the success of the center's overall mission. Even a center fortunate enough to begin with a large financial award must be continually thinking about attracting new sources of financial support for its programs or for the center as a whole if the center will continue beyond its initial funding. In addition, a center must have a clearly defined development plan and someone with the skills to carry it out. Since most scientists are not trained or skilled at development, center leaders should consider hiring someone who can fulfill this critical need. Identifying sources of funding outside the typical granting agencies, much less creating a picture of the center that attracts these sources, can be daunting. Our center leaders initially approached our member institutions that benefit most directly from the presence of the CBN, for financial support. Thus far, Georgia State University, the lead institution of the CBN, has agreed to provide up to 50% of the financial support required to maintain critical staff for the CBN after its NSF funding ends in 2009. Thus, a base of financial and local support is in place to promote the future of the center. This has been a critical first step. Nonetheless, without funding for our programs there will be little need for staff. Thus, finding funding for our programs remains a huge challenge for our center. We are currently developing creative methods that can be used to attract funding from outside sources. For example, each of the center's institutions already has in place a development office with trained professionals. The center leaders have begun working with those offices and their staff to identify and obtain financial support that will benefit the center, as well as the institutions, themselves. In addition, center leaders are forging a relationship with a regional business consortium whose mission could be enhanced by the promotion of the CBN. This consortium could potentially attract leading business and industry in the region to support the center and its mission. Using the research strengths developed by the center we hope to attract specific foundations with a desire to promote research in a particular field, such as understanding and developing treatments for Alzheimer's disease or autism. Moreover, there are a number of foundations, both regional and national, whose missions include the promotion of science education. These foundations might provide sources of funding for our educational programs. No doubt, finding sources of funding to continue the work currently being done in the CBN will be a huge challenge and will likely require that the center leaders reinvent some, if not all, aspects of the CBN and its mission. Moreover, a substantial change in the mission of the center could alienate some of our current members and supporters. Although no one wants funding to dictate the type of research and education we engage in, the fact is that funding is the foundation upon which most of the center rests and these are risks that we must face in light of that fact.

In conclusion, this review of the Center for Behavioral Neuroscience covers some of the potential challenges and issues that might arise during the design, start-up and maintenance of a multi-institutional, multi-disciplinary research and education center.
